# In the Chair: How to Guide Groups and Manage Meetings

**DOI:** 10.1192/pb.bp.115.052514

**Published:** 2016-06

**Authors:** D. D. R. Williams

My introduction to ward meetings at Claybury Hospital and departmental and staff meetings during my psychiatric training were negative experiences. I soon came to the view that these activities were largely unproductive and best avoided. In contrast, as a young consultant I soon realised that to enjoy a full professional life, participating effectively in, influencing the outcome of and chairing meetings were essential skills. I acquired several standard texts on committee work and the art of being a good chairman but they were not very useful. The bulk of their contents were about rules of etiquette and procedure based on the British parliamentary system. This model is now arcane and outmoded as the Westminster bubble remains in a time warp, while the world outside has moved on so that now committees and meetings are conducted in a more informal and relaxed way. In addition, these old texts had few useful tips about how to manage a big problem in committees – the difficult disruptive individual with an axe to grind.

Andrew Green's new book is very welcome. It is an excellent guide to those who perform a very important but underrated role of chairing groups and committees. Furthermore, it deals with how groups, committees and meetings should be run and how individuals can participate effectively. The author is well placed to write this book. From 1998 to 2013 he was head librarian and chief executive of the National Library of Wales, one of the five legal deposit libraries in the UK and a major archive repository. He has acted as chair of a large number of voluntary, public sector and official bodies. He has also been able to observe at first hand the technique of others chairing committees and meetings. To be a member of a committee with a superb chair can be a real pleasure and I regret that for me this did not occur more often.

The book deals in detail with the responsibilities of the chair for planning meetings, the task of chairing a board of a public body or company and the relationship between the chair and the chief executive. It also covers annual meetings, appointment panels and those quasi-judicial meetings: disciplinary and grievance hearings. Green is good on the two sections dealing with the mechanics and dynamics of conducting meetings. He places great emphasis on thorough preparation, the value of background work and of taking up issues with individuals outside the meeting itself. There are useful tips about how to steer a meeting through a period of turbulence and how to deal with the awkward committee member who starts up, ‘with great respect Mr Chairman’, clearly bristling for a fight.

It is a practical, comprehensive guide with a good index and references for further reading. I would strongly recommend it as essential reading for a young consultant psychiatrist who wants to establish a sound foundation for an important component of a rewarding professional career. In addition, it acts as a manual to be kept at hand for guidance when one is asked to accept a new responsibility such as attending an advisory appointments committee. Recently, it was mooted that I would have to participate in a telephone conference call about my long-term research project. This would be a new experience for me and I was glad to dip into this book for some helpful advice.

